# Mediterranean Diet Food Components as Possible Adjuvant Therapies to Counteract Breast and Prostate Cancer Progression to Bone Metastasis

**DOI:** 10.3390/biom11091336

**Published:** 2021-09-09

**Authors:** Paola Maroni, Paola Bendinelli, Alessandro Fulgenzi, Anita Ferraretto

**Affiliations:** 1 Laboratory of Experimental Biochemistry & Molecular Biology, IRCCS Istituto Ortopedico Galeazzi, Via R. Galeazzi 4, 20161 Milano, Italy; paola.maroni@grupposandonato.it (P.M.); anita.ferraretto@unimi.it (A.F.); 2 Dipartimento di Scienze Biomediche per la Salute, Università degli Studi di Milano, Via L. Mangiagalli 31, 20133 Milano, Italy; alessandro.fulgenzi@unimi.it

**Keywords:** Mediterranean diet, nutrients, bone metastasis, epithelial-mesenchymal transition, osteolytic bone metastasis, osteoblastic bone metastasis, breast cancer, prostate cancer

## Abstract

Bone metastasis is a serious and often lethal complication of particularly frequent carcinomas, such as breast and prostate cancers, which not only reduces survival but also worsens the patients’ quality of life. Therefore, it is important to find new and/or additional therapeutic possibilities that can counteract the colonization of bone tissue. High adherence to the Mediterranean diet (MD) is effective in the prevention of cancer and improves cancer patients’ health, thus, here, we considered its impact on bone metastasis. We highlighted some molecular events relevant for the development of a metastatic phenotype in cancer cells and the alterations of physiological bone remodeling, which occur during skeleton colonization. We then considered those natural compounds present in MD foods with a recognized role to inhibit or reverse the metastatic process both in in vivo and in vitro systems, and we reported the identified mechanisms of action. The knowledge of this bioactivity by the dietary components of the MD, together with its wide access to all people, could help not only to maintain healthy status but also to improve the quality of life of patients with bone metastases.

## 1. Introduction

Cancer is a potentially fatal disease that afflicts the whole population; the International Agency for Research on Cancer (IARC) estimates that approximately 18/19 million new cases are diagnosed each year and more than 50% of these will develop metastasis. The most dramatic aspect of cancer is represented by the phenomenon of metastatic spread, which is believed to be responsible for over 90% of deaths related to the neoplastic disease.

Data for breast cancer show that it continues to be the most frequent type of cancer in women. In fact, in 2020, the IARC estimated 2,261,419 cases worldwide (https://www.iarc.who.int/, accessed on 20 July 2021), in particular, Western lifestyle and obesity contribute to increasing the incidence of breast cancer [1]. Even if the improvement of therapies and the application of screening programs lead to an increase in survival for localized breast cancer, when patients develop metastasis, the average survival time after the onset of bone metastasis is 12–53 months [2]. IARC data for prostate carcinoma, the most common male cancer, estimated 1,414,259 cases in 2020 worldwide. Thanks to therapies, 84% of patients survive for ten years or more, but the percentage drops to 30% in metastatic prostate cancers [3]. Breast and prostate cancers display an inclination to metastasize to bone, with an incidence of 73% and 68%, respectively [4]. Skeleton metastases are also reported for thyroid cancer (60%), lung cancer (30%), bladder cancer (40%), renal cancer (20–25%), and melanoma (14–45%) [5]. As a whole, more than 80% of bone metastases derive from the breast, prostate, and lung. Furthermore, in advanced cancers, the increased survival, due to the improvement of therapies, is correlated to an augmented possibility to develop metastases in the skeleton [6,7], thus the incidence of bone metastases is expected to increase in the coming years.

Patients who develop bone metastasis often show clinical complications [5], the so-called skeletal-related events (SREs), such as bone pain, pathological fractures, spinal cord compression, and hypercalcemia. SREs cause the loss of mobility, compromise the structural integrity of the bone, reduce the quality of life, and reduce the overall survival. Sometimes, surgery is necessary to treat pathological fractures or nerve compression.

In recent years, studies have broadened knowledge of the biology of bone metastases and have offered new therapeutic opportunities such as systemic chemotherapy and radiotherapy, bisphosphonate, and anti-receptor activator of nuclear factor-κB (NF-κB) ligand (RANKL) antibody, but these treatments are not without side effects, such as osteonecrosis in the case of bisphosphonate. For this reason, and despite advances in the therapeutic approaches, bone metastasis remains largely incurable, and treatment of metastatic cancer still represents an open challenge for oncologists. In this context, there is a strong need to discover new and/or additional anti-metastatic therapies to improve patients’ outcomes.

The positive impact of lifestyle and nutrition on cancer incidence has already been fully reported [8] and recommendations to both prevent cancer and improve cancer patients’ health have been given by the World Cancer Research Fund in the continuous update progress (CUP, https://www.wcrf.org/diet-and-cancer, accessed on 15 February 2021). A healthy lifestyle characterized by the consumption of specific food categories together with physical activity and smoking and alcohol reduction can decrease the incidence of all cancer types. These recommendations constitute the basis of the Mediterranean diet (MD), which is characterized by plenty of cereals, fruits and vegetables, legumes, fish, nuts, and olive oil and a lower frequency of dairy food, red meat, and wine consumption. It is well known that a high adherence to the MD is associated with low cancer incidence, and this positive influence extends to cancer survivors [9,10], indicating that foods and/or specific nutrients could effectively synergistically act with pharmacological therapies or they could partly counteract the negative aspects of the disease, ameliorating the patients’ life [11].

Breast and prostate cancer are examples of neoplastic disease for which diet, and in particular the MD, before and after diagnosis, can influence the prognosis [1,12,13,14,15], although not all studies agree on real and positive outcomes.

The interpretation of data coming from studies conducted on patients diagnosed with breast/prostate cancer and with bone metastasis in reference to the MD suffers due to the methodologies used, that is, (i) the methods to determine the adherence degree to the MD; (ii) the time interval before and after cancer diagnosis in relation to the followed diet; (iii) the time interval before and after cancer progression to metastasis in relation to the followed diet; (iv) the lack of absolute certainty that the questionnaires used to investigate the diet quality could report true answers; (v) the interaction of the different nutrients and molecules associated in a meal. To overcome these problematic aspects and the limitation of the studies concerning the effect of single nutrients [11], the present review firstly highlights the process and pathways used by breast and prostate cancer cells to metastasize and, secondly, considers how nutrients within the MD could influence these specific processes. The match between chemio/radio therapies and specific nutrients could constitute a non-chemical approach free from side effects for the prevention of the growth of breast and prostate cancers. Lastly, due to its high-quality nutrient content, the MD can act as adjuvant therapy to alleviate suffering caused by SREs and improve the quality of life in bone metastatic patients.

## 2. Methods

PubMed was used to perform all the bibliographical searches among the publications in the English language by considering recent ones (from the last ten years) when a large amount of data was available, or without time limitations with a few publications. We used keywords related to different steps of the bone metastatic process, to molecular events occurring in bone tissue in different types of bone metastases, and we intersected them with biomolecules in the MD foods. We considered only bone metastasis derived from breast and prostate carcinomas. In detail, the following keywords and/or combinations of keywords were used: foods AND breast cancer within the last ten years; nutrients AND bone metastasis; epithelial-mesenchymal plasticity AND nutrients; invasiveness AND Mediterranean diet; foods AND osteoblastic bone metastasis; anthocyanins AND bone metastasis; nutrients AND osteoblastic bone metastasis; skeleton colonization AND nutrients; vimentin AND nutrients AND breast cancer; EMT AND nutrients; osteolytic bone metastasis AND nutrients. Chosen papers are reported in the reference list together with papers needed to explain the background of the topic.

## 3. Bone Metastasis: A Multi-Step Process

Several interconnected events are required to form a bone metastasis. To reach the new growth site, cancer cells must carry out a set of steps, known as the “metastatic cascade”, in which each passage requires the acquisition of specific biological properties by the tumor cells, such as the capacity to detach from the primary site, to move and infiltrate the surrounding tissue (epithelial-mesenchymal transition, EMT), to invade the vasculature, to survive in the circulation, to extravasate, and to colonize the new growth site. All these abilities belong to the metastatic cell phenotype.

### 3.1. Epithelial-Mesenchymal Transition: A Process That Regulates Invasiveness

EMT is a process in which cells lose their epithelial features (cell-cell adhesions, cell polarity, and differentiating characteristics) to acquire mesenchymal-like features, an event that in cancer is correlated to invasion, metastasis, tumor stemness, and resistance to therapy [16]. For epithelial tissue-derived tumors, EMT is decisive for tumor progression [17]. Morphological changes in the cells occur during EMT: cells acquire a spindle shape by decreasing the expression of cell adhesion molecules (i.e., E-cadherin) and by increasing the expression of mesenchymal markers (i.e., N-cadherin, fibronectin, and vimentin). Cancer cells with mesenchymal phenotype are present at the invasion front of different types of carcinomas, as in breast and prostate cancers [18]. The reverse process, named mesenchymal-epithelial transition (MET), occurs at metastatic sites. The reversibility of the phenotype testifies the plasticity of cancer cells, a feature that allows the cells to also assume intermediate phenotypes in the transition from one state to another, therefore gaining the most useful functional characteristics for their colonization path: the invasiveness and the resistance to anoikis for mesenchymal cells, the ability to grow in the new site for epithelial cells.

The EMT program is coordinated by transcription factors, including Snail1/Snail, Snail2/Slug, Twist, ZEB1, and ZEB2, whose relevant roles in cancer cells have been well established [14,19]. In carcinomas, the activation of the EMT program is triggered by Snail, which acts as a repressor of the transcription of epithelial markers, while the maintenance of invasive features is due to Twist and ZEB 1/2 activities [20].

The upregulation of mesenchymal markers leads to the remodeling of the cytoskeleton, an altered expression profile of the adhesion molecules, and the activation of matrix metalloproteinases (MMPs), such as MMP-2 and MMP-9. Motility and invasiveness of cancer cells are indeed associated with the destruction of extracellular matrix (ECM), an event due to the MMPs, which cleave molecules of the matrix, including basal membrane components. MMPs play a relevant role in invasion and metastasis, as demonstrated by numerous studies [21].

Several growth factors and cytokines, emanating from tumor stroma, are involved in the induction of the EMT program and favor the metastatic process. Among them, transforming growth factor beta (TGF-β) and hepatocyte growth factor (HGF) are particularly relevant in the progression of carcinomas to bone metastasis. TGF-β, through the downstream Smad pathway, appears as the most powerful EMT inducer [22]. Upon binding of TGF-β to the receptor, phosphorylated Smad2 and Smad3 complex Smad4 and translocate into the nucleus to upregulate EMT transcription factors [23]. Furthermore, TGF-β induces the EMT program through Smad-independent pathways, such as the Ras/Raf/Erk kinase [24], phosphatidylinositol 3-kinase (PI3K)/Akt/mTOR, tumor necrosis factor receptor-associated factor 6 (TRAF6)/TGF-β-activated kinase 1 (TAK1), and Wnt/β-catenin signaling pathways [25,26,27]. The HGF/Met receptor axis plays many roles in tumor progression, such as proliferation, evasion of apoptosis, invasion, and angiogenesis. Several signaling pathways are activated downstream of Met, including Ras/Raf/Erk, PI3K/Akt, and Wnt/β catenin and exert pro-tumorigenic roles. The HGF/Met axis also appears as a relevant inducer of EMT through the regulation of EMT-related transcription factors [28].

### 3.2. Skeleton Colonization: Alteration of Physiological Bone Remodeling

Bone is an attractive site for tumor colonization due to the wealth of calcium and the availability of stored growth factors, such as TGF-β, which is produced by osteoblasts, deposed in the bone matrix, and released during bone resorption [29]. Moreover, the so-called “metastatic niche” [30], a microenvironment created by primary tumors for future metastasis, in the case of bone, includes several cellular types. Bone endothelial cells, hematopoietic stem cells, and cells that compose the endosteal niche (osteoblasts, osteoclasts, and fibroblasts) characterize the bone milieu and condition the tumor growth [31]. Other cell types such as adipocytes, osteocytes, immune cells, and megakaryocytes are able to regulate the metastatic growth in the bone [32,33,34]. Furthermore, a plethora of signals, cytokines, and growth factors allow communication throughout the cells, creating the tumor-stroma crosstalk.

Upon arrival in the bone, cancer cells alter tissue homeostasis, which is finely tuned by the coordinate actions of several cell types, and this imbalance favors the development of secondary growth. It is possible to differentiate the bone metastases into two types, osteolytic and osteoblastic. Breast, lung, and renal cancers prevalently lead to bone destruction (osteolytic metastasis), whereas prostate cancer typically leads to bone-forming injury (osteoblastic metastasis). However, breast cancer can also develop mixed bone metastases, in which the skeletal lesions are characterized by active bone resorption and new bone formation, and osteolysis can occur in the metastases derived from prostate carcinomas [35].

After the interaction between tumor cells and bone cells, a positive feedback loop, the so-called vicious cycle, occurs. This process is characterized by the release of a series of cytokines and growth factors by cancer cells that affect the bone tissue; changes in the microenvironment result in the release of growth factors, which in turn feed the tumor, modifying its behavior and growth.

As regards tumor-stroma crosstalk in bone metastasis, the HGF/c-Met receptor axis and TGF-β appear to play relevant roles. HGF, as a stromal factor, finds its receptor Met on carcinoma cells and on osteoblasts and osteoclasts. Bone metastatic lesions derived from prostate cancers express high levels of Met, which are inversely correlated with the androgen receptor levels, and therefore related to the progression of the disease [36]. In a xenograft model of bone metastasis from breast cancer, HGF, more available in the bone microenvironment with respect to control bone, activates β-catenin signaling in cancer cells [37,38]. Of note, Met is also highly expressed in human bone metastatic tissue from breast carcinoma [39]. In the xenograft model of bone metastasis from breast cancer, the blockade of the HGF/Met axis or TGF-β prolongs the survival of animals [40].

#### 3.2.1. Osteolytic Bone Metastasis

High activity of the osteoclasts, accompanied by a reduced functionality of the osteoblasts, characterizes the osteolytic metastases. Several factors released by breast, lung, and renal carcinoma cells, such as parathyroid hormone-related protein (PTHrP), cytokines, and prostaglandins, are responsible for the osteoclast formation and activation with the consequent degradation of the bone matrix [41]. PTHrP and IL-11 act through the enhanced production of RANKL, which stimulates the differentiation of precursors of osteoclasts, leading to bone resorption [42]. PTHrP also reduces the expression of the RANKL antagonist osteoprotegerin (OPG), sustaining osteoclast formation [43]. TGF-β also participates in the induction of PTHrP expression in metastatic breast cancer cells [44].

Jagged1, a potent downstream mediator of TGF-β, is released during bone destruction and it was reported to function as an important mediator of bone metastasis by activating the Notch pathway in bone cells. Jagged1, by stimulating IL-6 release from osteoblasts and activating osteoclast differentiation, promotes tumor growth [45].

Tumor cells are also able to inhibit the activity of osteoblasts by the modulation of the Wnt signaling pathway, which is frequently deregulated in cancer. This pathway modulates a variety of cellular processes and is essential for osteoblast differentiation. The canonical β-catenin-dependent Wnt pathway stabilizes β-catenin, allowing the nuclear translocation of the protein. This event results in the activation of T cell factor/lymphoid enhancing factor (TCF/LEF) transcription and the expression of the target genes [46]. In breast cancer, the development of osteolytic metastasis involves the deregulation of Wnt agonists together with the expression of Wnt antagonists. In particular, the release of Dickkopf-1 (Dkk-1), a Wnt antagonist, by cancer cells, leads to the inhibition of osteoblasts and stimulation of osteoclasts [47]. Several other Wnt antagonists as well as bone morphogenetic protein (BMP) antagonists play a relevant role in the modulation of osteoblast differentiation/proliferation, thus contributing to bone destruction [48].

#### 3.2.2. Osteoblastic Bone Metastasis

Osteoblastic bone metastases are defined by their osteosclerotic appearance on X-rays. The deposition of new bone tissue drives the balance between bone resorption and bone formation in favor of the latter. Cancer cells release factors that activate osteoblasts to proliferate and form bone matrix.

ET-1 appears as the most important activator of osteoblasts, and is also able to inhibit the activity and motility of osteoclasts [49,50].

The binding of ET-1 to its receptor A (endothelin A receptor, ETAR) downregulates the autocrine production of a Dkk-1. As consequence, the Wnt pathway is activated, resulting in differentiation and function of osteoblasts [49]. Moreover, Wnt agonists are released by prostate cancers cells [51]: Wnt 3A, through the β-catenin pathway, leads to the expression of BMP-4 and BMP-6 in cancer cells, resulting in the promotion of osteoblastic lesions [52]. Other Wnt agonists, such as Wnt7B, activate osteoblasts through a non-canonical pathway, playing a relevant role in the osteogenic lesions in advanced prostate cancer [53]. BMPs released by cancer cells, besides stimulating osteoblasts, drive osteomimicry, a process by which cancer cells or bone microenvironmental cells assume an osteoblastic-like phenotype; tumor cells and endothelial cells express bone-specific proteins and participate in the deposition of new tissue [54]. Osteomimicry favors the interaction of metastasis with bone [55] and it is orchestrated by Runt-domain transcription factor 2 (Runx2), the key regulator of osteoblast differentiation. Indeed, in prostate carcinoma cells, Runx2 is expressed and correlates with metastatic potential [56], while in breast carcinoma cells Runx2 increases with respect to normal mammary tissue and regulates the expression of osteoblast-related genes [55].

Prostate cancer cells also produce Dkk-1 and inhibitors of BMPs, so the phenotype of bone metastasis will be dictated by the equilibrium between factors aimed at the bone formation and antagonists of osteoblast activation [57,58]. These complex interactions give rise to osteoblasts with a dysfunctional phenotype and the formation of a poorly compacted bone, characterized by disorganized type I collagen fibrils and osteoblasts not aligned along the collagen matrix. Mineralization is also aberrant due to the availability of phosphate and calcium, leading to a hypermineralized matrix. Overall, these events reduce bone strength and function [59,60].

## 4. Nutrients and Bone Metastasis

### 4.1. Nutrients in the Epithelial-Mesenchymal Plasticity

Many data have been collected regarding the ability of compounds mostly belonging to vegetables and fruits present in the MD to interfere with the phenotypic plasticity and invasiveness of different types of malignant cells, as asserted by in vitro studies, even if the mechanisms of action have not always been defined. Some of these compounds owe their anti-metastatic effectiveness to their ability to interfere with the EMT, downregulate the expression of MMPs, and act by modifying the behavior of signaling proteins. Concerning breast cancer, the most commonly used cellular system employs the triple negative and highly invasive MDA-MB-231 cells exposed to natural compounds.

It was reported that both polyphenols and non-phenolic nutrients are able to exert anti-invasive activity in vitro in many cell types, among the ones in breast cancer cells. *Resveratrol* has shown the ability to upregulate E-cadherin and downregulate mesenchymal markers in several cell types, including breast cancer cells, multiple myeloma cell lines, and colorectal cancer cells [61,62,63]. Resveratrol in breast cancer cells appears to increase E-cadherin expression through the inhibition of TGF-β1-induced EMT, by regulating Smad-dependent and Smad-independent pathways [59].

Resveratrol also prevents prostate cancer invasion and metastasis through several mechanisms: by reverting the EMT process, downregulating the androgen receptor and CXCR4 (CXCL12 chemokine receptor 4) pathway [64], by intervening in the bi-directional interplay between stromal and epithelial cells, and by inhibiting HGF-induced migratory behavior of prostate cancer cells [65]. Therefore, all these data reveal how resveratrol can intervene in the regulation of the metastatic phenotype of both breast and prostate cancer cells through multiple mechanisms.

Li et al. suggested that in prostate cancer cells, resveratrol can inhibit the EMT process (LPS induced, used to trigger EMT in PC-3 cells) probably through the inhibition of Hedgehog signaling, one of the pathways that regulates EMT in tumor growth [66].

*S-allylcysteine* (*SAC*) and *S-allylmercaptocysteine* (*SAMC*) present in garlic have shown the ability to restore E-cadherin expression in addition to inhibiting cancer cell proliferation in androgen-independent prostate cancer cells. The restoration of E-cadherin expression seems to be due to the contemporary transcriptional activation of the E-cadherin gene and the decreased expression of the Snail gene, an E-cadherin suppressor [67]. Another allium derivative, *diallyl disulfide* (*DADS*), has been demonstrated to both decrease the expression and protein synthesis and inhibit the activity of MMP-2 and -9 in human prostate carcinoma LNCaP cells [68]. In MDA-MB-231 breast cancer cells, DADS, besides similar effects on MMP-9, showed other abilities: inhibition of cell migration and invasion, reversion of EMT, and finally induction of apoptosis through the modulation of the β-catenin signaling pathway [69].

The tyrosol derivatives *(-)-oleocanthal* in extra virgin olive oil are recognized to act in more than one way in the modulation of the phenotype plasticity of mammary cancer cells: (i) suppressing the expression of the mesenchymal marker vimentin; (ii) restoring the expression of E-cadherin and Zo-1 in MDA-MB-231 cancer cells; (iii) stabilizing the expression of E-cadherin and Zo-1 in MCF-7 and BT-474 breast cancer cells; (iv) blocking the HGF/c-Met activation in MDA-MB-231, MCF-7, and BT474 cancer cell lines in culture [70,71].

*Kaempferol*, a phytoestrogen present in fruits and vegetables, is considered as a promising therapeutic agent for cancer metastasis for its action against adhesion, migration, and invasion of MDA-MB-231 human breast carcinoma cells [72]. In addition, kaempferol can reduce both the activity and expression of MMP-2, MMP-9, and cathepsin in MCF-7 breast cancer cell lines [72,73]. Moreover, in triple-negative breast cancer cells (TNBCs), low doses of kaempferol can downregulate RhoA and Rac1 signaling pathways. In ER-positive breast cancer cells (MCF-7), kaempferol, through the regulation of protein expression involved in EMT as well as the metastasis-related genes, suppresses E2 (17-β-estradiol) or triclosan-induced EMT, migration, and invasion [73,74]. Therefore, kaempferol could be considered as a potent chemopreventive compound against breast cancer metastasis, to be used alternatively to hormone replacement therapy.

*Indole-3-carbinol* (*I3C*) and *indole[3,2-b] carbazole* (*ICZ*) from cruciferous vegetables have shown the ability to inhibit breast cancer cell migration by multiple activities. First, in MCF-7 breast cancer cells treated with *I3C* and *ICZ*, E-cadherin mRNA expression was increased while vimentin mRNA expression was attenuated. Second, the MMP-2 and -9 activity as well as the focal adhesion kinase (FAK) mRNA expression were decreased, thus demonstrating an inhibition of the EMT process [75].

*Crocin* and *crocetin*, two saffron carotenoids, have displayed the ability to inhibit murine metastatic breast cancer cell (4T1) migration and invasion in addition to attenuating the adhesion to extracellular matrix in vitro. The mechanism affected seems to be related to the Wnt/β-catenin pathway [76].

*Soy isoflavones* are highly involved in the progression to bone metastasis of prostate cancer at different levels. The first level is the reversion of the EMT process. Low doses of *genistein* (*4′,5,7-trihydroxyisoflavone*) for 48 h are able to reverse the EMT process in prostate cancer cell lines LNCaP, LNCaP/HIF-1a, and IA8-ARCaP, as demonstrated by the cell morphological features and the upregulation of the E-cadherin together with the loss of expression of vimentin [77]. Genistein can also inhibit MMP-2 expression in both PC3 and LNCaP cells in a dose- and time-dependent manner [78]. In addition, genistein is able to diminish the expression of several MMP genes, especially of MMP-9, both in vitro (PC3 cells) and in vivo (PC3 bone metastasis xenograft model), presumably by inhibiting the NF-κB DNA-binding activity [79] involved in MMP gene expression [80].

*Anthocyanin 3,5-diglucoside*, the major phytochemical constituent of muscadine grape skin extract, due to its antioxidant potential, facilitated the MET process in prostate cancer cells characterized by re-expression of E-cadherin and reduced vimentin levels. This effect is due to the antagonization of the action of Snail, a key player of EMT, caused by the inhibition of the JAK/STAT pathway [81]. Since Snail overexpression increased cathepsin L activity via STAT3 signaling, useful for migration, invasion, and osteoclastogenesis, the muscadine grape skin extract, due to its content of anthocyanin, ellagic acid, and ellagic acid precursor, could be of interest as a therapeutic agent in breast and prostate bone metastasis [82].

*Silibinin*, a flavanone isolated from milk thistle, decreased vimentin protein expression in a dose- and time-dependent manner and suppressed MMP-2 expression, while it regulated cytokeratin-18 gene in ARCaPM cells, an in vitro model of prostate cancer progression to bone metastasis [83]. The possible mechanism seems to be related to the downregulation of Slug and ZEB1, EMT regulators [84].

*Curcumin*, one of three major curcuminoids derived from turmeric, is well known for a plethora of activities against tumor cells. As it regards the present issue, curcumin has shown the ability to (i) inhibit proliferation and colony formation of breast cancer cell lines, MCF-7 and MDA-MB-231; (ii) suppress the migration and invasion of MDA-MB-231 cells; (iii) downregulate the mRNA expression of vimentin, fibronectin, and β-catenin; (iv) upregulate the mRNA expression of E-cadherin. Together, these activities indicate the possibility to counteract the EMT process in breast tumors [85].

*Ferulic acid*, a polyphenol contained in numerous plants, can inhibit migration and revert EMT in vitro in MDA-MB-231 cells. This effect was also confirmed in vivo in the xenograft model of breast cancer [86].

**Figure 1:**
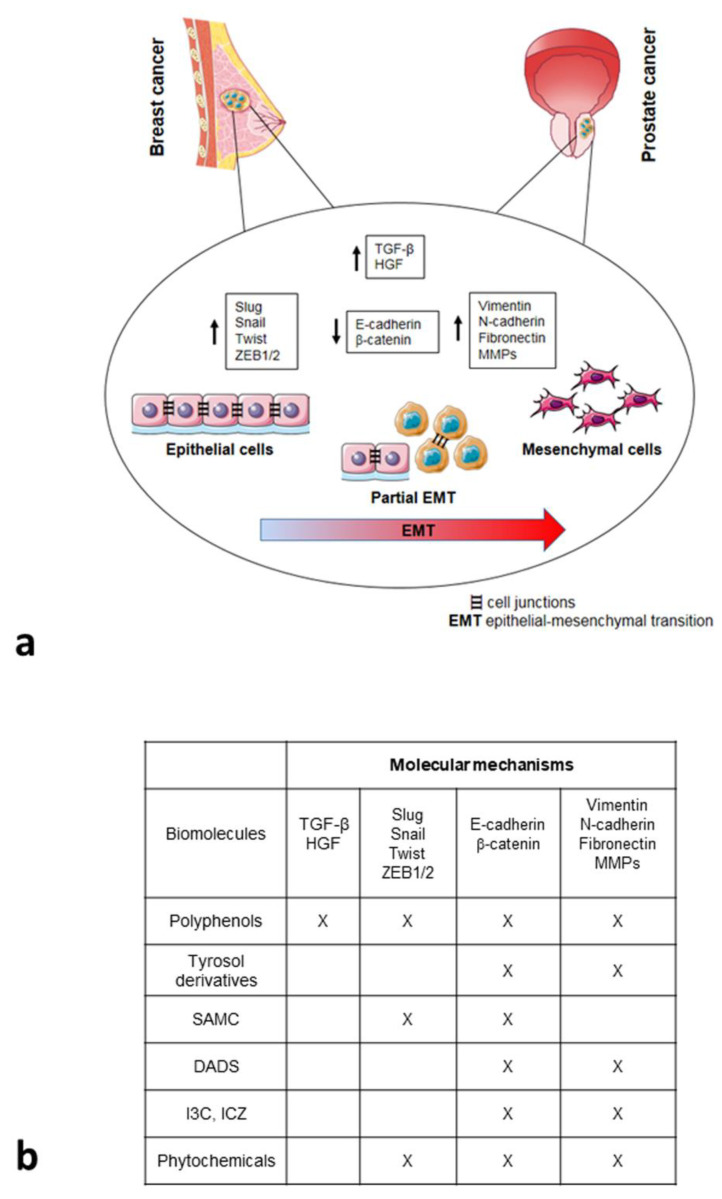
Epithelial-to-mesenchymal transition (EMT) program in breast and prostate cancers and roles exerted by different compounds belonging to Mediterranean diet. (**a**) Molecular processes leading to EMT development. MMPs, metalloproteinases; TGF-β, transforming growth factor beta; HGF, hepatocyte growth factor (figure created using Servier Medical Art available at https://smart.servier.com, accessed on 10 June 2021). (**b**) Bioactive molecules in EMT process grouped for chemical nature and their site of action, which is indicated by “X”.

Figure 1 summarizes all these data, indicating the intervention of different components of the MD in the phenotypic plasticity of metastatic cells.

### 4.2. Nutrients in the Osteolytic Bone Metastasis

The combined effect of dietary grape polyphenols (5 mg/kg each of resveratrol, *quercetin*, and *catechin*) was tested on the progression of mammary tumors in the highly metastatic ER (-) MDA-MB-435 cell line. Molecular analysis of excised tumors demonstrated that the treatment reduces tumor growth due to upregulation of forkhead box O1 (FOXO1) and NFKBIA (IκBα), thus activating apoptosis and potentially inhibiting NF-κB activity. The image analysis of distant metastases demonstrated that grape polyphenols reduce metastasis, especially to liver and bone [87]. Some turmeric extracts with a precise content of phenolic compounds have been demonstrated to inhibit MDA-MB-231 cell growth and the secretion of PTHrP that drives breast cancer bone metastases in advanced disease as an osteolytic factor [88,89]. In in vitro experiments using MDA-MB-231 cells, curcumin also revealed the ability to block Smad-dependent TGF-β signaling, which is supposed to induce PTHrP release [90] and the progression to bone metastasis in vivo [88]. Interestingly, successive experiments have demonstrated that curcumin-glucuronide is the prevalent circulating form and that bone marrow cells can carry out curcumin deglucuronidation. This ability at the bone level is of fundamental importance since the released curcumin is able to reduce the receptor-mediated phosphorylation of Smad2 [89]. Curcumin can also suppress RANKL-induced osteoclastogenesis induced by prostate cancer cells [91].

Green tea (Camellia sinensis) extract containing the polyphenols *epicatechin* (*EC*), *EC gallate* (*ECG*), *epigallocatechin* (*EGC*), and *EGC gallate* (*EGCG*) has demonstrated anti-metastatic and anti-osteolytic effects in in vitro and in vivo experiments using 4T1 cells, a mouse mammary tumor cell, and in a mouse mammary tumor model obtained by inoculating 4T1 cells at the subcutaneous level. The activities exerted by the extract are (i) the dose- and time-dependent inhibition of in vitro cell viability; (ii) the increase in the expression of the pro-apoptotic protein Bax; (iii) the decrease in the expression of the anti-apoptotic protein Bcl-2; (iv) the inhibition of the migration and invasion of 4T1 cells; (v) the decrease in the number of in vitro osteoclasts together with a decrease in osteolysis in vivo; (vi) an increase in the bone volume [92].

*Sulforaphane* is a phytochemical from cruciferous vegetables with known anti-cancer properties. As concerns the present issue, the main positive effect of sulforaphane treatment is the enrolment of a new gene network, i.e., RUNX2, NF-κB1, and SOX9, which becomes downregulated, and in turn negatively affects the transcription and secretion of collagen type 1 α1 (COL1A1), a metastasis-promoting factor, MMP-9, and cathepsin K (CTSK), matrix-degrading factors involved in breast cancer metastasis. In addition, sulforaphane inhibits osteoclast differentiation [93].

In a xenograft model of bone metastasis, Pore et al. demonstrated that the oral administration of sulforaphane reduced breast cancer-induced osteolytic bone metastasis via a significant decrease in circulating IL-8 [93].

*N*-3 polyunsaturated fatty acids such as *docosahexaenoic acid* (*DHA*) and *eicosapentaenoic acid* (*EPA*) contained in fish oil have been shown to prevent the formation of osteolytic lesions by targeting the pro-metastatic protein CD44, implying the suppression of metastases to the bone. In a model of bone metastasis of breast cancer, it was argued that fish oil is an important dietary supplement to consider in adjuvant therapy for bone metastases [94].

Other authors have reported that DHA attenuates breast cancer bone metastasis and associated osteolysis more potently than EPA, possibly by inhibiting migration of breast cancer cells to the bone as well as by inhibiting osteoclastic bone resorption [95].

It is worth remembering that bone loss due to the combined mechanisms of osteoclast activation and estrogen depletion can also occur during aromatase inhibitor therapy for breast cancer with a consequent increase in fracture rate and osteoporosis. Although not directly related to bone metastasis, the positive effects of *vitamin D* [96], EPA, and DHA supplementation [97] in reducing bone loss in these patients deserve to be considered.

*Trolox*, a vitamin E analog, has been shown to inhibit breast cancer-induced bone destruction when administered to mice before the injection of 4T1 breast cancer cells in an experimental model of osteolytic metastasis. The authors claim that trolox exerts anti-metastatic and anti-osteolytic activities in breast cancer cells through Prostaglandin E_2_ (PGE_2_)-dependent and PGE_2_-independent mechanisms [98].

*Glycitein*, a phytoestrogen belonging to the group of isoflavones, increased osteoclast apoptosis and decreased the mRNA expression of RANKL, without affecting OPG, in a murine in vitro model. Moreover, glycitein decreased IL-6 mRNA expression in osteoblasts. IL-6, a well-known pro-inflammatory cytokine, is involved in bone resorption and in osteoclast formation and thus the interesting role exerted by glycitein on IL-6 should be analyzed in more detail [99].

Dietary *genistein* was able to upregulate the expression of OPG in PC3 bone tumors, leading to a possible inhibitory effect on osteoclast formation [80]. Subsequent studies have reported that *daidzein* and genistein increased PTHrP and PTH type 1 receptor (PTH1R) expression in human PCa cell lines in addition to the OPG/RANKL protein ratio [100].

More recently, a supposed synergistic action by daidzein and genistein in a soybean extract was able to stimulate the secretion of OPG and inhibit that of RANKL, a critical transcription factor for osteoclast differentiation, in osteoblasts, thus producing an indirect but useful inhibition of osteoclast differentiation [101].

Although the exact mechanisms are not fully elucidated, it was reported that in a murine model of bone metastasis, the vitamin D deficiency can affect the vicious cycle, resulting in increased growth of breast cancer cells in the bone environment, accompanied by osteolytic lesions [102].

Resveratrol upregulates protein and mRNA expression of major histocompatibility complex class I chain-related proteins A and B (MICA and MICB) in breast cancer cells, which in turn promote breast cancer cell lysis by natural killer (NK) cells in vitro and in vivo. In this way, resveratrol could both counteract the immune escape and improve the immunogenicity of cancer cells [103].

**Figure 2:**
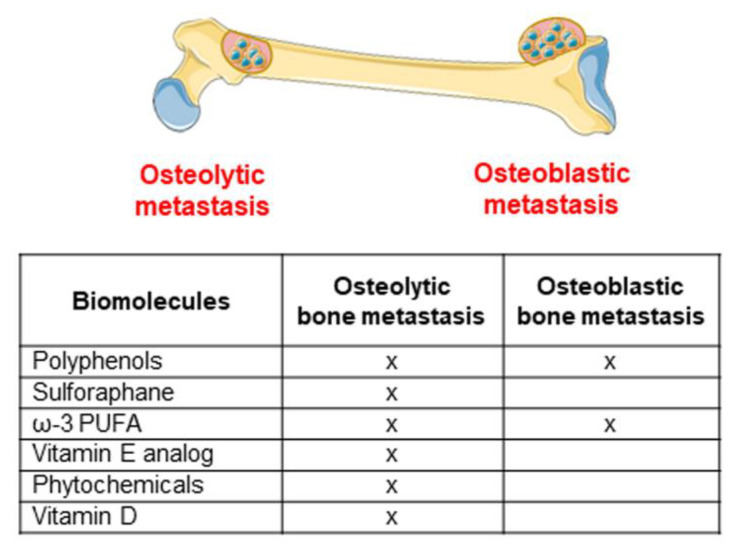
Dietary compounds involved in the inhibition of osteolytic and osteoblastic metastasis development (figure created using Servier Medical Art available at https://smart.servier.com, accessed on 10 June 2021). “X” indicates the involvement in the processes.

In Figure 2, bioactive molecules in the MD are reported for their role in the development of osteolytic and osteoblastic bone metastasis.

### 4.3. Nutrients in Osteoblastic Bone Metastasis

Genistein and soy isolate have demonstrated the ability to induce a possible decrease in Wnt/β-catenin expression and protein levels, respectively, by modulating GSK-3 activity through the frizzled 3 receptor, resulting in increased degradation of β-catenin and cell growth [13].

The flavonoid *p-hydroxycinnamic acid* (*HCA*) has demonstrated prevention activity toward suppressed osteoblastogenesis and enhanced osteoclastogenesis in MDA-MB-231 cells co-cultured with bone marrow cells. The supposed mechanism is the antagonization of the activation of NF-κB signaling induced by RANKL [104].

As concerns bone colonization by prostate cancer, curcumin has demonstrated multiple activities: it downregulates the expression of CXCR4, the receptor of the stromal chemokine CXCL12 expressed by osteoblasts and involved in the metastatic process [105]; it inhibits the serine/threonine kinase Akt activation and suppresses cell proliferation [106]. Moreover, curcumin can block the chemotactic effects of CC motif ligand 2 (CCL2) on invasion, adhesion, and motility of PCa cells; the effect is partially due to a differential regulation of PKC and MMP-9 signaling [107]. Dorai et al. analyzed the possibility of using curcumin as a therapeutic agent in advanced prostate cancer, particularly concerning skeletal complications. Curcumin seems to modulate TGF-β (that plays a central role in the vicious cycle of bone metastasis) through the antagonistic action exerted by BMP-7 in both osteolytic and osteoblastic metastasis from prostate cancer. Thus, curcumin is able (either directly in cancer cells or indirectly in bone marrow-derived stem cells) to reprogram the check and balance of TGF-β signaling pathways by the upregulation of the expression of BMP-7. Using an animal model of bone metastasis, the authors argued the importance of using curcumin as a dietary ingredient to prevent bone metastasis [108].

It has been reported that dietary intake of ω-*3 PUFAs* decreases the risk of developing aggressive/metastatic prostate carcinoma [109]. Brown and colleagues reported that high ω-3: ω-6 PUFA ratios together with a large amount of eicosapentaenoic acid (EPA) in the diet can counteract the metastatic process to bone through the blocking of PGE_2_ production, leading to a reduced risk of aggressive disease [110].

In Figure 2, bioactive molecules in the MD are reported according to their role in the development of osteoblastic metastasis.

### 4.4. Nutrients with an Assessed Anti-Bone Metastatic Role although Not Specifically Related to Breast/Prostate Cancer or to the Mechanisms Described Above

There are a plenty of studies highlighting the ability of different natural compounds, mainly present in vegetables, to inhibit or counteract the formation and progression of bone metastasis. Although these studies are not directly related to breast/prostate cancer, it is of interest to consider them in the perspective that some of these compounds and activities could in the future also be associated with prostate/breast cancer and bone metastasis.

*Dietary N-(4-hydroxyphenyl) retinamide* (*4-HPR*), a synthetic amide of retinoic acid, has demonstrated anti-metastatic effects in the highly aggressive in vivo mouse prostate reconstitution (MPR) model in which either heterozygote or homozygote p53-deficient fetal prostate is initiated with ras and myc oncogenes. Multiple pathways associated with cell apoptosis and/or G1 arrest seem to be involved in the *4-HPR* activity [111].

The activation of silent information regulator 7 (SIRT7) deacetylase by resveratrol inhibits breast cancer lung metastasis by antagonizing TGF-β1 signaling [12].

The flavonoid *apigenin (API),4′,5,7-trihydroxyflavone* can suppress the oncogene Sparc/osteonectin, cwcv, and kazal-like domains proteoglycan 1 (SPOCK1) expression normally upregulated in prostate cancer and responsible for the invasion and metastasis of cancer cells in a human prostate cancer xenograft model. The exact mechanism used by API consists in targeting the Snail/Slug-mediated EMT process [112]. In addition, API can suppress the signaling pathway due to IL-6 which is responsible for chronic inflammation associated with breast cancer, for instance, and responsible for the EMT process, the invasion and migration of tumor cells [113].

Table 1 summarizes the nutrients and related foods with a defined role as fighters against the formation of bone metastasis.

## 5. Conclusions

The importance of a balanced and safe diet is well known in reference to the onset of diseases, including chronic diseases, such as cardiovascular diseases, diabetes, and overweight/obesity, but much work has to be done to fully realize the possibility to efficiently couple medicine and foods in order not only to prevent disease but also to ameliorate patients’ lives. From this point of view, cancer and metastasis represent a large field of interest due to the high mortality and costs of care. The Mediterranean diet is recognized as a dietary pattern with many positive results and, above all, it is accessible to all people of all ages, thus efforts are to be made to explore its components and relative bioactivity. In the present review, the biomolecules contained in MD foods are reviewed for their ability to inhibit, reverse, or block metastasis to bone, a condition that deeply and negatively affects life and survival. Many of these biomolecules derive from plants and are characterized by poor solubility and bioavailability and, sometimes, after metabolic transformation, they do not retain the anti-metastatic properties. All these problems make it difficult to employ these biomolecules and successive studies are needed to understand how to overcome these complications. Some examples are present in the literature, for instance curcumin-loaded nanoparticles, which through multiple pathways inhibit the growth of prostate cancer cells both in vitro and in vivo [114], or structurally modified curcumin, which overcomes the above limitations [115]. Moreover, for plant-derived biomolecules the seasonal and regional variation should be considered. The MD not only consists of plant-derived foods but, in fact, EPA and DHA, as well as vitamin D, also possess anti-metastatic bone bioactivity and, for this reason, the MD can offer the possibility to combine different biomolecules and thus different therapeutic approaches. The presence of the biomolecules, here considered, in foods largely consumed in the MD like cereals, vegetables, olive oil, fish, and fruits (Table 1), indicates the need to choose and combine different everyday meals to reach and maintain a healthy status.

To date, clinical trials that consider the MD as an adjuvant therapy in patients with bone metastases have not been reported in the literature. Notwithstanding, a few interesting papers could indicate the positive effects of the MD on the progression of breast carcinoma. For example, it has been reported that the MD may contribute to reducing breast cancer recurrence in patients with invasive breast cancer [116], and that adherence to the MD in breast cancer survivors is associated with a better quality of life [117]. Lastly, a pilot study has reported that the supplementation of vitamin D in women with metastatic breast cancer and insufficient 25-hydroxyvitamin D (25[OH]D) serum levels improves bone pain and fatigue [118]. Together, the studies reported here highlight the necessity to follow this line of research.

## Figures and Tables

**Table 1:** Biomolecules selected in the present discussion, foods that contain them, and references that report their role in countering processes involved in bone metastasis.

Biomolecules	Foods	References
resveratrol	grapes, red wine, peanuts, berries	[59,61,62,63,64,65,66,103]
s-allylcysteine (SAC) s-allylmercaptocysteine (SAMC)	broccoli, Brussels sprouts, cauliflowers	[67]
diallyl disulfide (DADS)	garlic	[68,69]
(-)-oleocanthal	extra virgin olive oil	[70,71]
kaempferol	leafy vegetables, apples, onions, broccoli, berries, tea, cabbage, endive, kale, beans, tomato, strawberries, leeks, grapes	[72,73,74]
indole-3-carbinol (I3C) indole[3,2-b] carbazole (ICZ)	cauliflower, cabbage, kale, garden cress, bok choy, broccoli, Brussels sprouts, mustard plants, leafy vegetables	[75]
crocin crocetin	saffron	[76]
genistein (4′,5,7-trihydroxyisoflavone)	soy	[77,78,79,80]
anthocyanin 3,5-diglucosides	berries, currants, grapes, tropical fruits, leafy vegetables, grains, roots, tubers	[81,82]
silibinin	milk thistle	[84]
curcumin	curry powder	[85,88,89,90,91,105,106,107,108,114,115]
ferulic acid	rice, wheat, oats, pineapple, grapefruit, orange, banana, berries, vegetables, flowers, leaves, beans, coffee beans, artichoke, peanut, nuts	[86]
quercetin	kale, tomatoes, broccoli, blueberries, apples	[87]
catechin epicatechin (EC) ec gallate (ECG) epigallocatechin (EGC) egc gallate (EGCG)	red wine, chocolate, tea, almonds, apples, blackberries, fava beans, hazelnuts, pistachios, plums, raspberries, strawberries	[92]
sulforaphane	cabbage, cauliflower, Brussels sprouts, bok choy, kale, collards, mustard greens, watercress	[93]
docosahexaenoic acid (DHA) eicosapentaenoic acid (EPA)	salmon, foraging fish, shellfish, tuna, walnuts, sardines, herring, mackerel, halibut	[94,95,97,109,110]
vitamin D	tuna, mackerel, salmon, cheese, egg yolks	[96,102]
trolox (vitamin E derivative)	wheat germ oil, sunflower seeds, almonds, sunflower oil, hazelnuts, peanut butter, corn oil, spinach, broccoli, soybean oil, kiwi fruit, mango, tomato, spinach	[98]
glycitein	soy and soy products,	[99]
daidzein and genistein	soy and soy products	[100,101]
*p*-hydroxycinnamic acid (HCA)	wasabi leafstalk, coffee, tea, wine, apples, berries, plums, cherries, peaches, citrus fruits, carrots, salad, cabbage, eggplant, artichoke, cereals, grapes	[104]
*n*-(4-hydroxyphenyl) retinamide (4-HPR)	synthetic retinoid	[111]
apigenin (API)	parsley, celery, celeriac, chamomile tea	[112,113]

## Data Availability

Not applicable.
